# Detection of glucosamine as a marker for *Aspergillus niger*: a potential screening method for fungal infections

**DOI:** 10.1007/s00216-021-03225-7

**Published:** 2021-02-22

**Authors:** Christopher L. Allison, Alex Moskaluk, Sue VandeWoude, Melissa M. Reynolds

**Affiliations:** 1 Department of Chemistry, Colorado State University, 1801 Campus Delivery, Fort Collins, CO 80523 USA; 2 Department of Microbiology, Immunology, and Pathology, Colorado State University, 1601 Campus Delivery, Fort Collins, CO 80523 USA; 3 Department of Chemical and Biological Engineering, Colorado State University, 1370 Campus Delivery, Fort Collins, CO 80523 USA; 4 School of Biomedical Engineering, Colorado State University, 1376 Campus Delivery, Fort Collins, CO 80523 USA

**Keywords:** *Aspergillus*, LC-MS, Chitin, Glucosamine

## Abstract

Several species of fungus from the genus *Aspergillus* are implicated in pulmonary infections in immunocompromised patients. Broad screening methods for fungal infections are desirable, as cultures require a considerable amount of time to provide results. Herein, we developed degradation and detection methods to produce and detect D-glucosamine (GlcN) from *Aspergillus niger*, a species of filamentous fungus*.* Ultimately, these techniques hold the potential to contribute to the diagnosis of pulmonary fungal infections in immunocompromised patients. In the following studies, we produced GlcN from fungal-derived chitin to serve as a marker for *Aspergillus niger*. To accomplish this, *A. niger* cells were lysed and subjected to a hydrochloric acid degradation protocol. Products were isolated, reconstituted in aqueous solutions, and analyzed using hydrophilic interaction liquid chromatography (HILIC) in tandem with electrospray ionization time-of-flight mass spectrometry. Our results indicated that GlcN was produced from *A. niger*. To validate these results, products obtained via fungal degradation were compared to products obtained from the degradation of two chitin polymers. The observed retention times and mass spectral extractions provided a two-step validation confirming that GlcN was produced from fungal-derived chitin. Our studies qualitatively illustrate that GlcN can be produced from *A. niger*; applying these methods to a more diverse range of fungi offers the potential to render a broad screening method for fungal detection pertinent to diagnosis of fungal infections.

**Supplementary Information:**

The online version contains supplementary material available at 10.1007/s00216-021-03225-7.

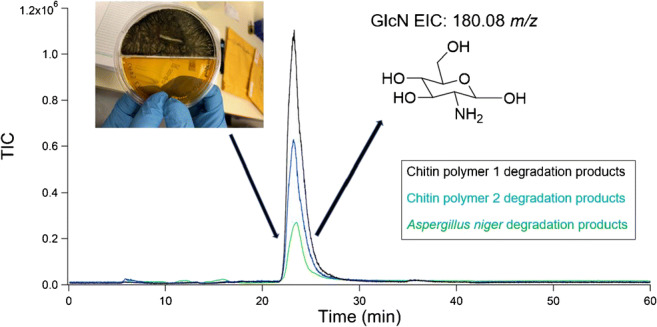

## Introduction

*Aspergillus niger* is a ubiquitous species of filamentous fungi [1, 2]. Colony formation can be visualized after a few days of growth by characteristic black coloration, which is produced by conidial spores [3]. *A. niger* is a species of filamentous fungus which produces branching hyphae [4] and is widely studied for its ability to produce citric acid, along with industrially significant enzymes such as glucoamylase [5–7]. In addition, it is a well-known plant pathogen [8], and is responsible for black mold formation which can lead to food spoilage in human food sources such as onions and grapes [5]. Occasionally, *A. niger* can be an opportunistic human pathogen [9]. Inhalation of spores can lead to pulmonary aspergillosis, which usually occurs in immunocompromised patients [10–12]. Diagnosis of respiratory aspergillosis remains problematic, leading to delays in treatment and high mortality rates [13]. One of the primary structural constituents of filamentous fungi is chitin, a β-1-4-linked homopolymer of GlcNAc residues [14]. In the following studies, D-glucosamine (GlcN) produced from structural chitin in *A. niger* and used as a molecular indicator for the presence of fungus. Ultimately, these methods hold the potential to screen for the presence of filamentous fungi, and play a role in fungal infection diagnostics.

Chitin is a β-1-4-linked homopolymer of GlcNAc residues [14]. It is the second most abundant natural polysaccharide in the world, behind cellulose [15, 16]. Chitin is a component of crustacean and arthropod exoskeletons and is found in fungal cell walls, where it confers rigidity to its parent organism [17]. It exists in nature as nearly straight microfibrils with average diameters of ~ 2.8 nm and of indeterminate lengths [18]. Its size has been reported from ~ 100 GlcNAc residues in yeast to 5–8000 residues in crab exoskeletons [19]. While most species of yeasts contain only 1–2% chitin, its abundance in filamentous fungi ranges from 10 to 30% [14]. Chitin is found in the cell walls and septa of all species of pathogenic fungi [20]. Some fungi produce chitin deacetylase enzymes that modify chitin to chitosan during biosynthesis; however, chitosan’s abundance in most fungi has not been well-defined [14, 21, 22].

**Fig. 1:**
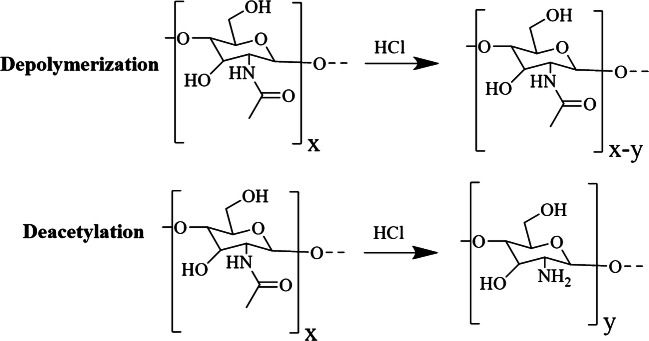
Simultaneous chemical modifications that occur upon the exposure of chitin to HCl. Polymers are simultaneously depolymerized and deacetylated, producing a low molecular weight chemical fingerprint that consists primarily of GlcN

Chitin is ubiquitous in the cell walls of fungi but is not found endogenously in humans. While GlcN can be found endogenously, the introduction of an aqueous extraction prior to the production of chitin-derived GlcN would effectively remove residual species, as chitin is insoluble prior to modification. In the following experiments, we tested fungal-derived chitin degraded from *Aspergillus niger*, a filamentous fungal species closely related to *A. fumigatus*, and characterized its degradation products using LC-MS. Upon exposure to HCl, chitin is simultaneously depolymerized and deacetylated as shown in Fig. 1, resulting in GlcN monomers and oligomers that can be related back to the presence of the parent polymer. These residues are soluble in aqueous liquids and can be separated and detected using LC-MS. Our research indicates that chitin-derived GlcN can be produced from *A. niger*, a species that has been implicated in fungal pulmonary infections. To accomplish this, we obtained *A. niger* cells and developed sample preparation steps to separate extracellular and intracellular components. We subjected fungi to cell lysis and degradation protocols, then used LC-MS to characterize the degradation products. We found that GlcN was quickly produced from chitin via simultaneous depolymerization and deacetylation of these intracellular polysaccharides.

Fewer than 1% of fungal species are potentially pathogenic [23]. Fungal spores are found ubiquitously in the environment, and most individuals are exposed to large numbers of airborne fungi with no notable health repercussions. However, a minority of fungal species are extremely effective opportunistic pathogens [24]. Worldwide, over 300 million cases of serious fungal infections occur annually [25]. Fungal infections can occur superficially (e.g., athlete’s foot) or as systemic infections such as candidemia or aspergillosis [26–29]. Pulmonary fungal infections comprise a significant number of systemics fungal diseases, with global incidence of > 10,000,000 patients. 1,400,000 of these result in fatalities annually [25]. A variety of genera such as *Aspergillus*, *Cryptococcus*, and *Pneumocystis* are responsible for these infections [30]. Chronic pulmonary aspergillosis is listed by The Global Action Fund for Fungal Infections (GAFFI) as one of four “priority fungal infections.” Disease is most often caused by *Aspergillus fumigatus* but can also be caused by *A. niger* or *A. flavus* [30]. Chronic pulmonary aspergillosis (CPA) is most frequently seen in patients that have at one time had lung disease, such as tuberculosis, chronic obstructive pulmonary disease, or lung cancer [31].

CPA progresses rapidly in immunocompromised patients, resulting in high mortality rates and necessitating prompt treatment [32, 33]. We propose that liquid chromatography-mass spectrometry (LC-MS) can be used as a broad screening method to assist in diagnosing pulmonary fungal infections (e.g., CPA) via the detection of chitin contained within fungal cell walls. Sputum or lavage samples from patients with pulmonary fungal infections contain fungi [34, 35], facilitating noninvasive sample acquisition. Chitin may be detected via the separation and detection of GlcN monomers and oligomers that are produced during the controlled degradation of chitin. Given this, detecting polymeric chitin has the potential to serve as a screening method for pulmonary fungal infections in clinical settings. Our results are significant as they corroborate methods of chemical modification and instrumental detection which can be used in tandem to detect fungal-derived GlcN. Our instrument configuration was chosen to provide a straightforward qualitative analysis that fungal-derived GlcN can be produced from the chemical modification of *A. niger* and can subsequently be detected using LC-MS. Having established the efficacy of our methods, we suggest that future experiments approaching clinical applicability test our methods using an LC-MS configuration common in clinical laboratories, such as an LC-MS/MS. Eventually, we hope that these methods will be implemented clinically as part of fungal infection diagnostics.

## Materials and methods

### Materials

*A. niger* fungi were obtained from domestic cat hair samples and cultured on an agar medium. D-Glucosamine hydrochloride was obtained from MP Biomedicals (Santa Ana, CA). Chitin polymer (100% acetylated) and *N-*acetyl-D-glucosamine (> 98.0%) were obtained from TCI (Portland, OR). Chitin polymer (20–30% deacetylated) was obtained from Alfa Aesar. ACS-grade acetone and Optima LC-MS grade formic acid were obtained from Fisher Chemical (Waltham, MA). Hydrochloric acid was obtained from Ward’s Science (Rochester, NY). LC-MS grade water, LC-MS grade acetonitrile, low molecular weight chitosan (96% deacetylated), and ammonium acetate (> 98.0%) were obtained were obtained from EMD Millipore (Burlington, MA). 0.2-μm Captiva Econofilters were obtained from Agilent (Palo Alto, CA). C18 Macro spin columns were obtained from Harvard Apparatus (Holliston, MA).

### Sample preparation

*A. niger* was grown and maintained on Sabouraud dextrose agar at 22 °C until harvesting. The fungal culture was originally obtained from domestic cat hair sample and cultured on a dermatophyte test media (DTM) agar plate via standard toothbrush method. The identification of the fungi was performed using colony morphology and Internal Transcribed Spacer 2 (ITS-2) region sequencing. To obtain ITS-2 sequencing results, fungus was harvested for DNA extraction and used for conventional PCR analysis. PCR products were sent for sequencing at Psomagen and sequences were compared to entries in GenBank. *A. niger* cells were transferred into 15-mL centrifuge tubes in a biosafety hood. Five milliliters chilled acetone (− 20 °C) was added prior to removal from the hood. Samples were incubated for 60 min at − 20 °C. Following incubation, samples were vortexed for 30 s then centrifuged for 10 min at 15 k×*g*. The supernatant was removed, and samples were allowed to stand for 30 min to encourage the evaporation of residual acetone. Liquid nitrogen was added directly to the dried pellets to lyse cells. After 5 min, 5 mL of − 20 °C acetone was added and the pellet vortexed again for 30 s. The supernatant was decanted, and the pellet was allowed to evaporate for 30 min. Four milliliters 10 M HCl was added to the dried pellet. The pellet was vortexed, and the resulting suspension was transferred to a round-bottom flask. The flask was heated in a water bath held at 90 °C and the acid evaporated over ~ 6 h. Following evaporation, the dried material in the flask was reconstituted using Milli-Q grade water (18.2 MΩ•cm). Aliquots were drawn from the flask and passed through 0.2-μm filters. The eluent was added to C18 spin columns and centrifuged for 4 min at 2 k×*g*. The filtrate was collected and analyzed via LC-MS.

### LC-MS methods

LC-MS analyses were carried out on an Agilent 6224 time-of-flight mass spectrometer coupled to an Agilent 1260 binary liquid chromatograph (Agilent, Palo Alto, CA). Separations were performed using a Thermo Fisher Acclaim HILIC-10 column with dimensions of 4.6 × 150 mm and 5-μm particle sizes. The mobile phase was composed of LC-MS grade water and acetonitrile (ACN). Each mobile phase component contained 10 mM ammonium acetate and 0.05% formic acid with a final pH 4.0. The total length of chromatography runs was 50 min. Solvent flow rate was set to 0.350 mL/min for the duration of the separation. A gradient elution was used with initial solvent proportions of 90:10 ACN:H_2_O. The solvent ratio was adjusted to 80:20 ACN:H_2_O from 0 to 30 min. From 30 to 31 min, the solvent ratio was returned to 90:10 ACN:H_2_O. From 31 to 50 min, the column was allowed to re-equilibrate and the baseline stabilize. The ion source used was a dual electrospray ionization source operating in positive ionization mode. Ion source conditions were as follows: 3500 V capillary voltage, 120 V fragmentor voltage, 60 V skimmer voltage, 250 V octupole voltage, 10 L min^−1^ gas flow (N_2_) at 300 °C, and 45 psig nebulizer pressure. The detection range was set to 95–3200 *m/z*.

### LC-MS of controls and samples

Chitin polymer 1 and chitin polymer 2 were subjugated to HCl degradation (see Table 1 for the specific properties of these polymers). For LC-MS analysis of polymer degradation products, ~ 4 mg chitin polymer 1 and chitin polymer were 2 were added to a 50-mL round-bottomed flask. An appropriate quantity of 10 M HCl was added to the powdered samples. The flask was heated in a water bath held at 90 °C and the acid evaporated over ~ 6 h. Following evaporation, the dried material in the flask was reconstituted using Milli-Q grade water (18.2 MΩ•cm). Aliquots were drawn from the flask and passed through 0.2-μm filters. The filtrate was collected and analyzed via LC-MS. All experiments were performed in triplicate.

**Table 1:** Polymers used for degradation studies

Polymer	Supplier	% GlcN	% GlcNAc
Chitin 1	Alfa Aesar	20–30^a^	70–80
Chitin 2	TCI	–	100^b^

^a^Alfa Aesar certificate of analysis; ^b^TCI certificate of analysis

### Data analysis

All data were analyzed using Agilent MassHunter Qualitative Analysis B.07.00 software. ESI optimization was performed prior to these experiments and a library of potential degradation products was generated to enable extracted ion chromatogram (EIC) scans for data deconvolution. Degradation experiments performed on *A. niger* samples were compared to the LC-MS results for chitin polymer degradations. Retention times and accurate mass measurements were both accounted for to provide a two-step validation confirming the identity of degradation products.

## Results and discussion

### Analysis of GlcN standards and chitin polymers

GlcN standards were purchased from MP Biomedicals. Standards were dissolved in LC-MS grade water to a concentration of 1 mg/mL and analyzed using LC-MS. Ions that were observed are listed in Table 2. Chromatograms and mass spectra associated with these results can be found in the Supplementary Information (ESM).

**Table 2:** Ions observed in MS extractions from LC-MS of GlcN standards

Signal observed (*m/z*)	Adduct
162	[C_6_H_13_NO_5_ - H_2_O + H]^+^
180	[C_6_H_13_NO_5_ + H]^+^
202	[C_6_H_13_NO_5_ + Na]^+^
381	[(2)C_6_H_13_NO_5_ + Na]^+^

While it was expected that the most prevalent ion would be a protonated molecule at 180 *m/z*, the most abundant ion in mass spectra was shown at 381 *m/z.* To the best of our knowledge, dimerization has not been previously reported in ESI analysis of GlcN; however, [2 M + H]^+^, [2 M + Na]^+^, and variations of these dimers have been observed in other compounds [36, 37]. Dimerized ESI ions are proposed to result from noncovalent interactions between residues in solution, as Coulombic barriers preclude dimerization following electrospray ionization [38]. One hundred sixty-two, 180, and 202 *m/z* are commonly seen ions in ESI analysis of GlcN. 162 *m/z* occurs following the dehydration and protonation of GlcN and has previously been observed in the ESI analysis of GlcN [39]. 180 *m/z* is a protonated GlcN molecule for which formation is promoted by the acidic pH in the mobile phase used. 202 *m/z* represents a sodiated adduct.

**Fig. 2:**
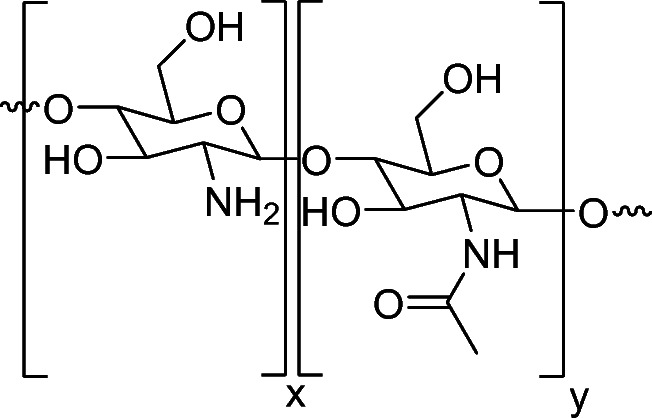
Generic structure of chitin (> 50% GlcNAc) showing GlcN (x) and GlcNAc (y) subunits

The efficacy of degradation methods was established via identification of the degradation products from chitin polymers following their exposure to HCl degradation protocols. In these experiments, two chitin polymers were exposed to HCl degradation protocols and the products were characterized using LC-MS. Information regarding the polymers used for degradation studies can be found in Table 1, along with a generic structure of chitin/chitosan shown in Fig. 2.

**Fig. 3:**
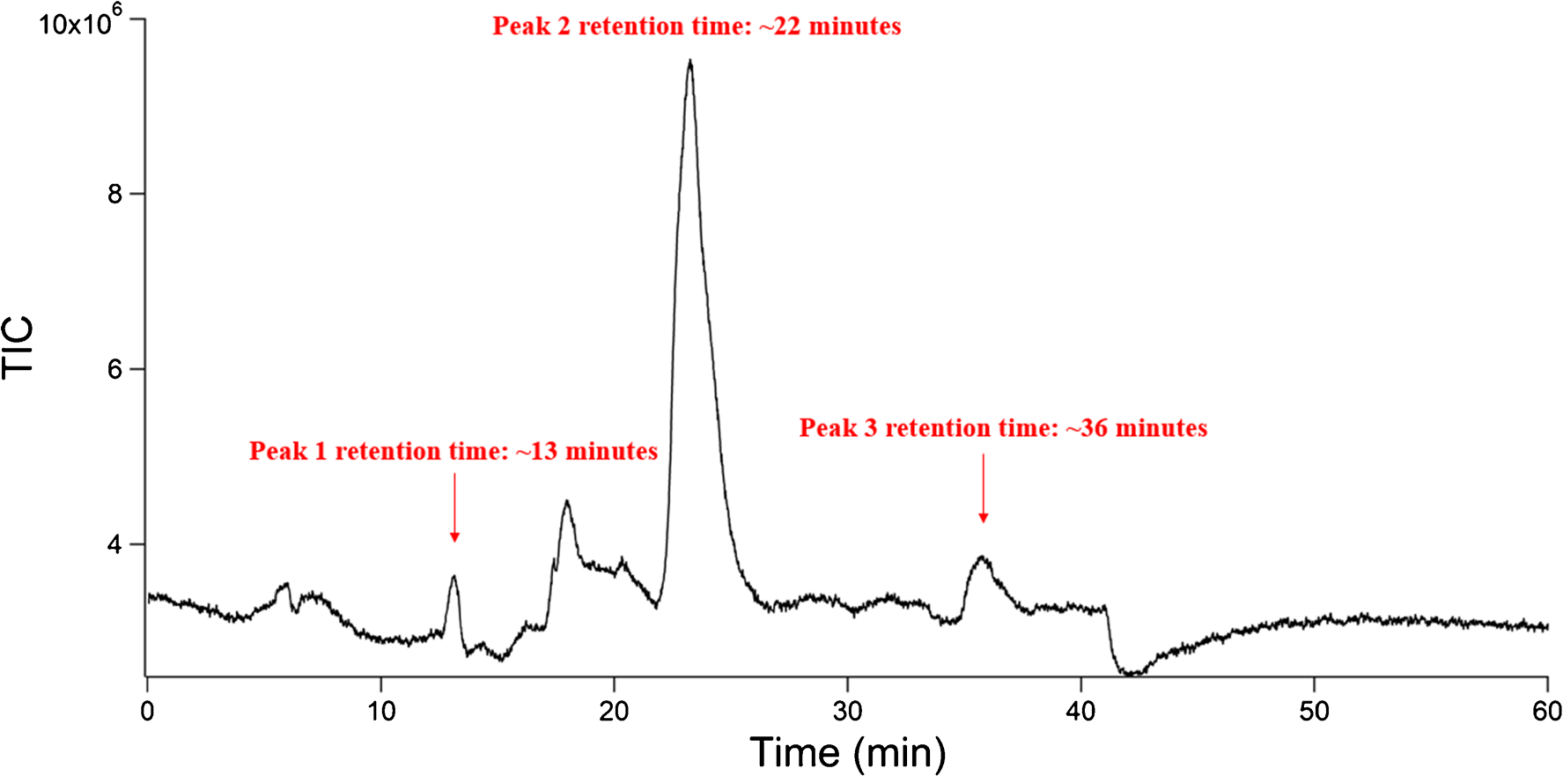
Representative chromatogram of chitin polymer 1 degradation products. Three peaks contained *m/z* that indicated the presence of the degradation products of chitin. Unlabeled peaks did not contain *m/z* that correlated to chitin degradation products

The first polymer used in these studies had a composition of ~ 75:25 GlcNAc to GlcN residues. This polymer was exposed to 5 M HCl at a concentration of 1 mg/mL in a round-bottom flask. The acid was removed under heat and evaporation over approximately 6 h. Milli-Q grade water (18.2 MΩ•cm) was added to the dry flask to resuspend soluble species. The resulting suspension was removed, filtered, and analyzed via LC-MS. The chromatogram obtained from these experiments is shown in Fig. 3.

Three peaks in the chromatograms obtained from these experiments contained *m/z* indicating the presence of the degradation products of chitin. The first of these peaks eluted at ~ 13 min and contained GlcNAc. The most prominent peak in this chromatogram eluted at ~ 24 min and contained GlcN. A third peak eluting at ~ 36 min contained GlcN dimer adducts. The peak containing GlcNAc was small in comparison to the GlcN peak, which was expected given the ability of HCl to deacetylate GlcNAc residues. Concentrated HCl depolymerizes chitin polymers considerably faster than it deacetylates them [39]. Hence, observation of acetylated species was not surprising. GlcN was predicted to be the prevalent product from this degradation protocol, given it is the final product of the depolymerization and deacetylation of chitin polymer. This prediction was confirmed by the relative size of the peak eluting at 24 min. Given the propensity of concentrated acid to deacetylate chitin more rapidly than it deacetylates GlcNAc residues, the size of this peak also indicated that depolymerization was largely complete by the time the HCl had evaporated. The peak at 36 min containing GlcN-dimerized adducts was relatively small in size and was representative of fully deacetylated, partially depolymerized residues. A summary of the peaks and the compounds eluting in these can be seen in Table 3. Mass spectral assignments for each peak can be found in the ESM.

**Table 3:** Summary of peaks and the ions contained within these

Retention time	Compounds	*m/z* identified
13 min	GlcNAc	204, 222, 244, 465
24 min	GlcN	162, 180, 202, 381
36 min	GlcN dimers	341, 363, 703

**Fig. 4:**
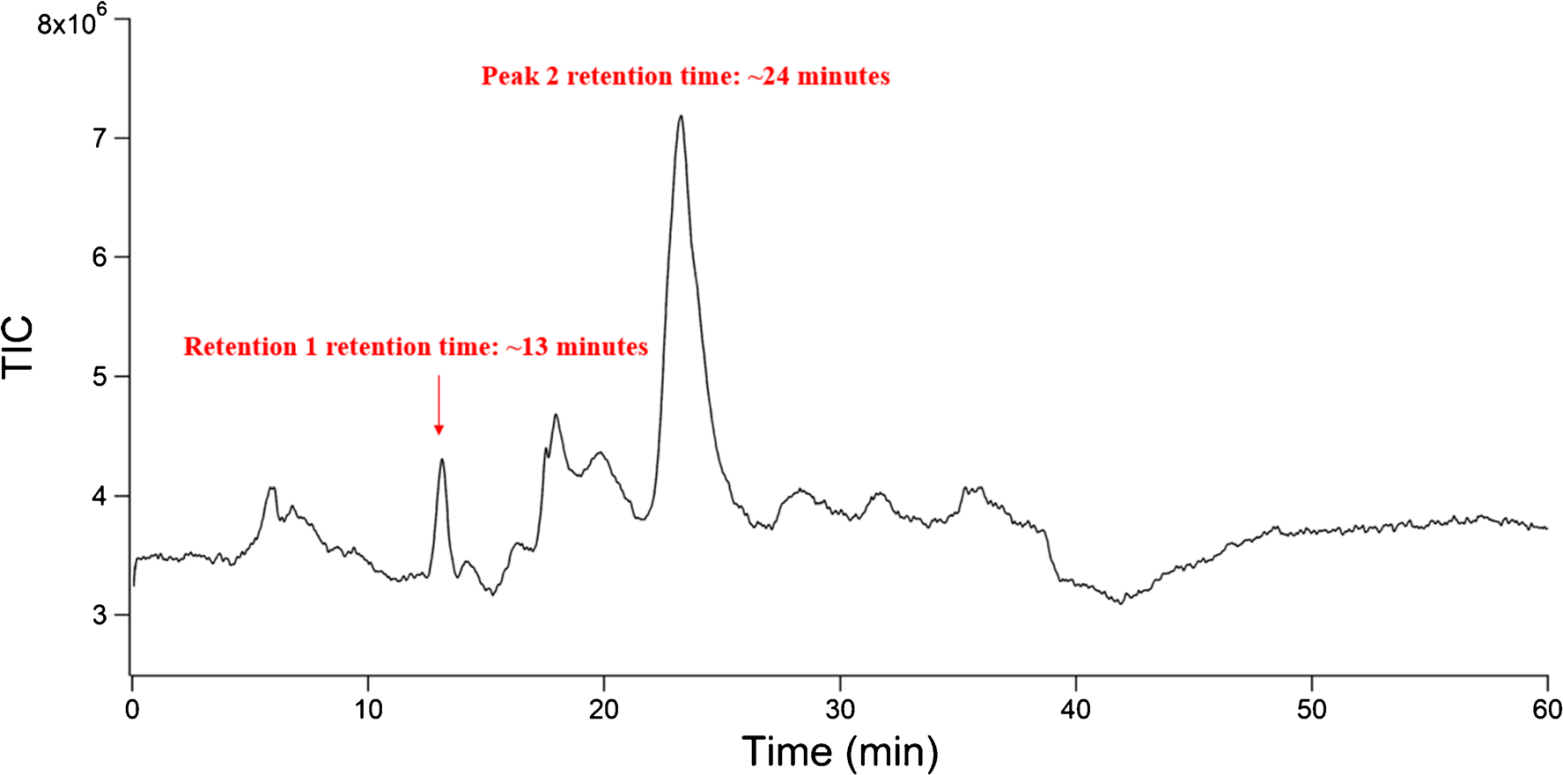
Chromatogram of chitin polymer 2 degradation products. Two peaks contained *m/z* that indicated the presence of chitin degradation products. Remaining peaks did not contain *m/z* representative of chitin degradation products

The second polymer used exposed to the HCl degradation protocol was chitin polymer 2, which had a composition of 100% GlcNAc. These studies mirrored those with chitin polymer 1, with this analyte being exposed to 10 M HCl at a concentration of 1 mg/mL in a round-bottomed flask. The acid was removed under heat by evaporation over approximately 6 h. Milli-Q grade water (18.2 MΩ•cm) was added to the dry flask to resuspend soluble species. The resulting suspension was removed, filtered, and analyzed via LC-MS. The chromatogram obtained from these experiments is shown in Fig. 4.

Two peaks in the chromatograms obtained from these experiments contained *m/z* representative of chitin’s degradation products. The first of these eluted at 13 min and contained GlcNAc. The second peak eluted at 24 min and contained GlcN. The peak at 13 min (containing GlcNAc) was small in comparison to the peak at 24 min (containing GlcN). Given the ability of HCl to deacetylate GlcNAc, we expected a lower quantity of GlcNAc to be present. These results were consistent with those from chitin polymer 1. A third peak at 36 min contained GlcN dimers; however, these results have not been included as the peak barely protruded above the baseline. A summary of the peaks and the compounds eluting in these can be seen in Table 4. Mass spectral assignments for both peaks can be found in the ESM.

**Table 4:** Summary of peaks and the ions contained within these

Retention time	Compounds	*m/z* identified
13 min	GlcNAc	204, 222, 244, 465
24 min	GlcN	162, 180, 202, 381

### Analysis of degradation products from *A. niger*

**Fig. 5:**
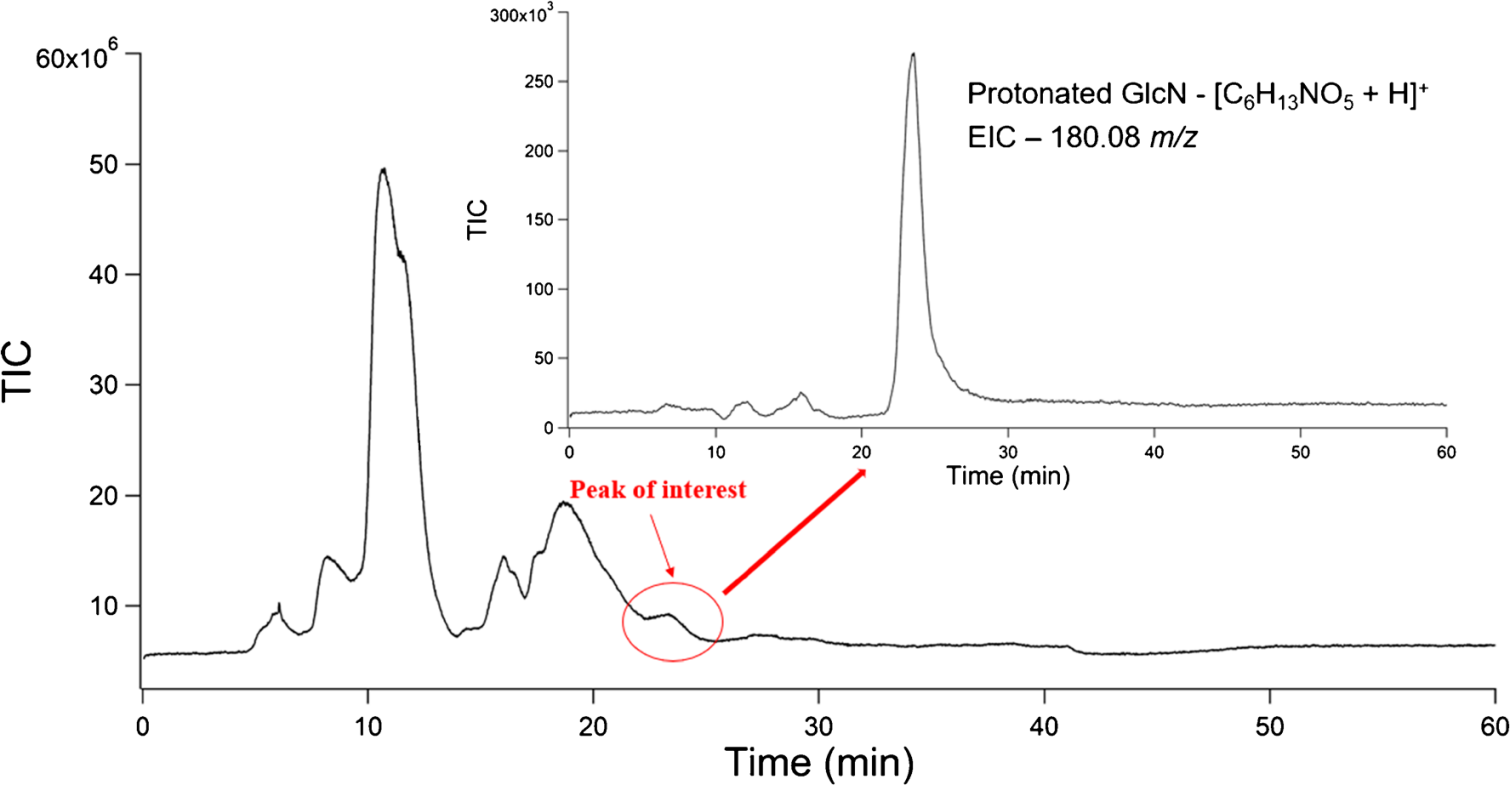
Chromatogram of *A. niger* degradation products. Analytes of interest eluted in a broad peak at ~ 24 min. The inset shows an extracted ion chromatogram for the monoisotopic mass of GlcN, 180.08

Following the testing of LC-MS methods using chitin polymers, the ability of degradation methods in tandem with LC-MS to detect fungal-derived GlcN was explored. *A. niger* cells were used for these studies. Cell lysis and precipitation steps were performed first, followed by solid-phase extraction steps for sample cleanup. Following this, we analyzed the degradation products using LC-MS. A representative chromatogram from these experiments can be seen in Fig. 5.

**Fig. 6:**
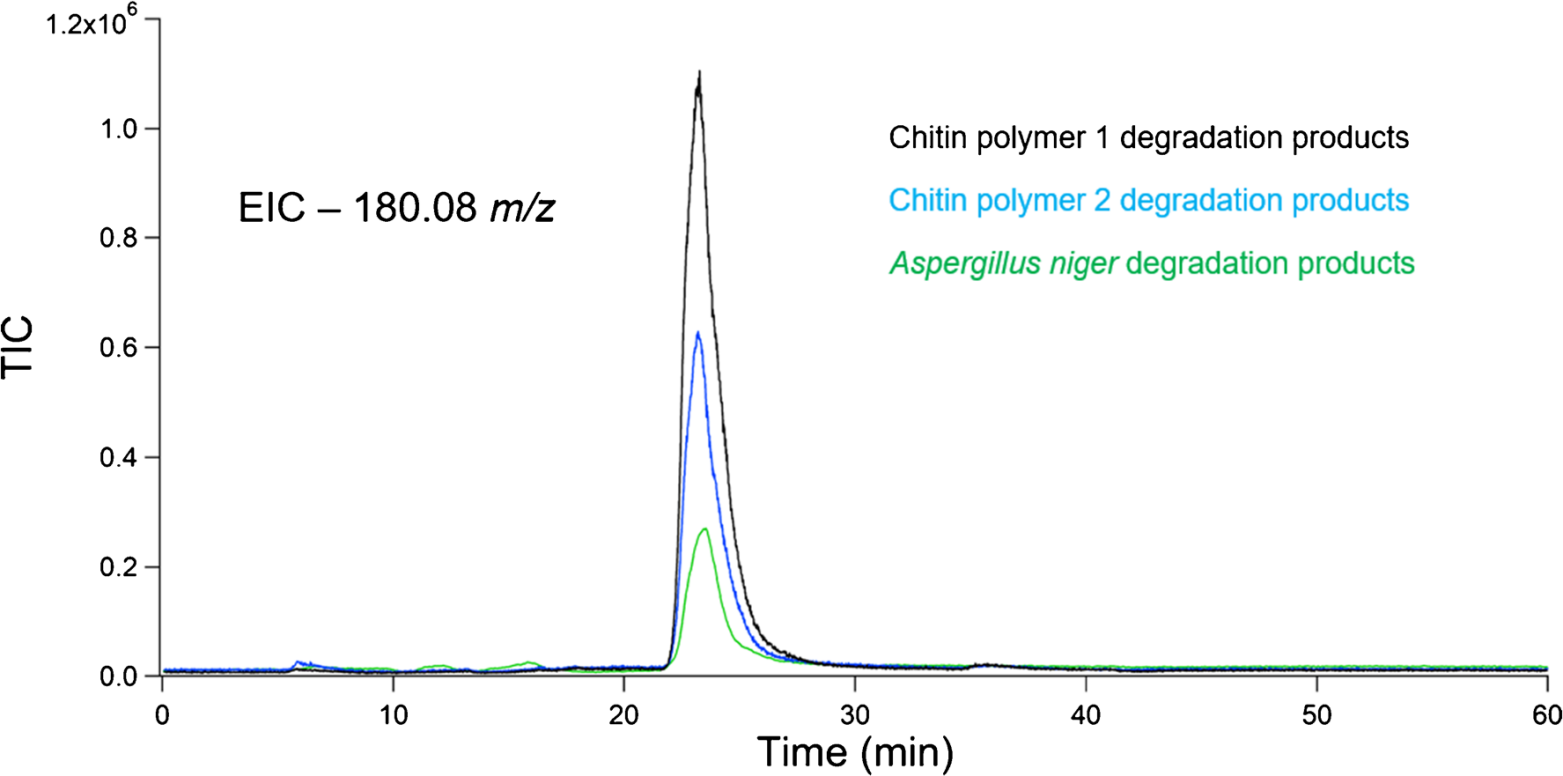
Overlay of EICs from degradation experiments. The value for EIC scans was set to 180.08 to highlight the elution of protonated GlcN. The black trace represents GlcN produced from chitin polymer 1. The blue trace represents GlcN produced from chitin polymer 2. The green trace represents GlcN produced from *A. niger* fungi

**Fig. 7:**
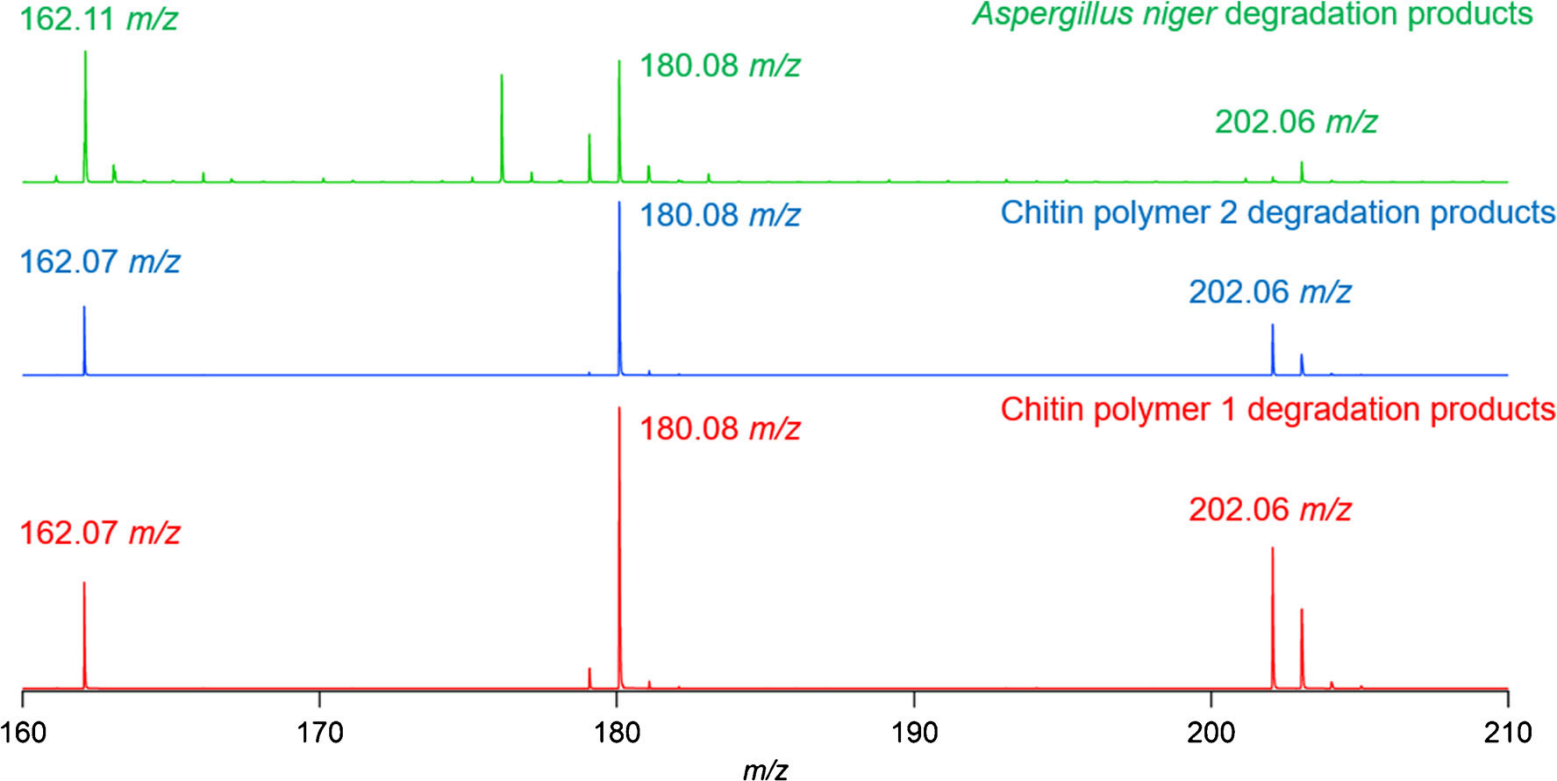
MS overlays from LC-MS analysis of polymer and fungal degradations. MS extractions from chitin polymer 1 are shown in black. MS extractions from chitin polymer 2 are shown in blue. MS extractions from *A. niger* are shown in green

The unedited chromatogram obtained from the extraction and degradation of chitin from *A. niger* contained multiple peaks that overlapped in areas from 5 to 25 min. As a HILIC HPLC column was used, it was inferred that compounds eluted from least hydrophilic to most hydrophilic. The final peak eluted at ~ 24 min and contained GlcN, along with *m/z* representing coeluting compounds. An extracted ion chromatogram (EIC) for the accurate mass *m/z* measurement of a protonated GlcN molecule was performed, and the peak was overlaid with those from chitin polymer degradation experiments. EICs were performed in this instance to specifically highlight the predicted degradation product from the degradation of chitin, and to validate that polymer models and fungi both produce GlcN during degradation. The EIC overlays confirm the production of GlcN from both chitin polymers studied, as well as the chitin contained in *A. niger* cells*.* The chromatographic peaks for both chitin polymers as well as fungal extractions line up at ~ 24 min. This overlay can be seen in Fig. 6. Mass spectra were extracted from these peaks, which can be found in Fig. 7. Chromatographic elution times and analysis of *m/z* values suggest the presence of ions with identical polarity and molecular weights, providing a two-step validation of the presence of GlcN in all samples tested.

Extractions of mass spectra from chromatographic peaks at ~ 24 min all displayed *m/z* at 180*,* indicative of ions with formulas matching those of protonated GlcN molecules, [C_6_H_13_NO_5_ + H]^+^. The degradation of both chitin polymers displayed a *m/z* at 202, indicative of ions with formulas matching those of sodiated GlcN adducts, [C_6_H_13_NO_5_ + Na]^+^. The mass spectrum of *A. niger* contained a *m/z* at 202; however, its abundance was very low. The degradation products from chitin polymers also displayed a *m/z* at 162, indicative of ions with formulas matching those of protonated dehydrated GlcN, [C_6_H_13_NO_5_ - H_2_O + H]^+^. While the mass spectrum of *A. niger* contained a *m/z* at 162, its monoisotopic mass varied from that expected from a protonated dehydrated GlcN ion. Closer examination revealed that the peak at 162.1109 *m/z* was split, suggesting that two molecules with similar but not identical *m/z* ratios were present in mass spectra. Greater resolving power would be required necessary to distinguish between these peaks.

Further studies to differentiate between endogenous and exogenous GlcN derived from chitin will be necessary prior to implementation of these methods in clinical facilities. Separating GlcN from these sources could be done using several approaches: by aqueous liquid extraction, by sub-micron filtration, or by dialysis. In addition, centrifugation has been shown to be an effective means of concentrating fungal spores [40]. Regardless of the approach chosen, a crucial aspect of separating endogenous and exogenous GlcN is that endogenous GlcN is removed prior to the production of GlcN from chitin, thereby reducing the likelihood of false positives. Use of an aqueous extraction step prior to the degradation of chitin would remove endogenous GlcN, given its high solubility. Alternatively, isolating fungi immediately following sample acquisition could be accomplished by sub-micron filtration, dialysis, or centrifugation and recovery of spores and other insoluble species prior to their exposure to HCl. The detection of GlcN by LC-MS/MS systems has been performed in multiple studies, with limits of quantitation at or below 10 ng/mL [41–43]. For our qualitative application, these values indicate limits of detection for GlcN in biologically relevant matrices as low as ~ 3 ng/mL for clinically relevant LC-MS systems. Ultimately, we hope that the methods presented herein may serve as a key component in the rapid detection of fungi.

## Conclusions

In our studies, we used LC-MS to detect GlcN from fungal-derived chitin. To accomplish this, *A. niger* fungi were obtained and exposed these to cell lysis and sample cleanup steps. Lysed cells were exposed to previously developed degradation protocols using HCl. The acidic solution was evaporated, and water-soluble analytes were resuspended into an aqueous suspension. These were filtered and analyzed using HILIC-ESI-MS. LC-MS analysis indicated the presence of a chromatographic peak with a retention time matching that of GlcN produced from the degradation of chitin. Mass spectral extractions of this peak provided secondary confirmation of its identity, showing a *m/z* at 180.08, matching that of a protonated GlcN molecule. Chitin degradation methods were developed to degrade fungal-derived chitin and to produce GlcN. The analyte produced from fungal degradations matched those generated during the analysis and comparison of degradation protocols using several variations of chitin and chitosan. Retention times and accurate mass measurements were compared to validate that chitosan polymers, chitin polymers, as well as *A. niger* produce GlcN following exposure to HCl. Cumulatively, the novelty of our results lies in our combination of chemical modification methods and analytical detection methods (HILIC-ESI-MS) to produce and detect GlcN from *A. niger*. By applying reproducible methods to detect a species of fungus implicated in pulmonary fungal infections, our studies address a clinical problem using sound analytical chemistry. Ultimately, we hope these methods will be implemented into clinical labs for purposes of fungal diagnostics.

## Supplementary Information

ESM 1 (DOCX 818 kb)

## Declarations

### Source of biological material

The source of cells used is covered in Section 2 Materials and methods under *Materials.*

### Statement on animal welfare

Hair collection from shelter-owned cats has been approved by Colorado State University’s Clinical Review Board and has been granted an Institutional Animal Care and Use Committee (IACUC) waiver. The veterinarians at the collaborating shelters have given consent for hair sample collection from their shelter-owned cats and performed the collections themselves.
