# Supplementary material for: Detection of glucosamine as a marker for Aspergillus niger: a potential screening method for fungal infections
Source: Anal Bioanal Chem. 2021 Feb 22;413(11):2933–41. doi: 10.1007/s00216-021-03225-7 (PMC8043943; doi:10.1007/s00216-021-03225-7)
Supplement: Supplementary file 1 — (DOCX 818 kb) [file 216_2021_3225_MOESM1_ESM.docx]

**Supplementary Information**

**Detection of glucosamine as a marker for *Aspergillus niger*: A potential screening method for fungal infections**

Christopher L. Allison^a^, Alex Moskaluk^d^, Sue VandeWoude^d^, and Melissa M. Reynolds^a,b,c^

^a^Department of Chemistry, Colorado State University, 1801 Campus Delivery, Fort Collins, Colorado 80523, USA

^b^Department of Chemical and Biological Engineering, Colorado State University, 1370 Campus Delivery, Fort Collins, Colorado 80523, USA

^c^School of Biomedical Engineering, Colorado State University, 1376 Campus Delivery, Fort Collins, Colorado 80523, USA

^d^Department of Microbiology, Immunology, and Pathology, Colorado State University, 1601 Campus Delivery, Fort Collins, Colorado 80523, USA

Contact information: Chris.Allison@colostate.edu, Melissa.Reynolds@colostate.edu, [alex.moskaluk@colostate.edu](mailto:alex.moskaluk@colostate.edu), Sue.Vandewoude@colostate.edu

Corresponding author: Melissa M. Reynolds

E-mail: Melissa.reynolds@colostate.edu

Telephone: (970) 491-3775

**LC-MS of GlcN standards**


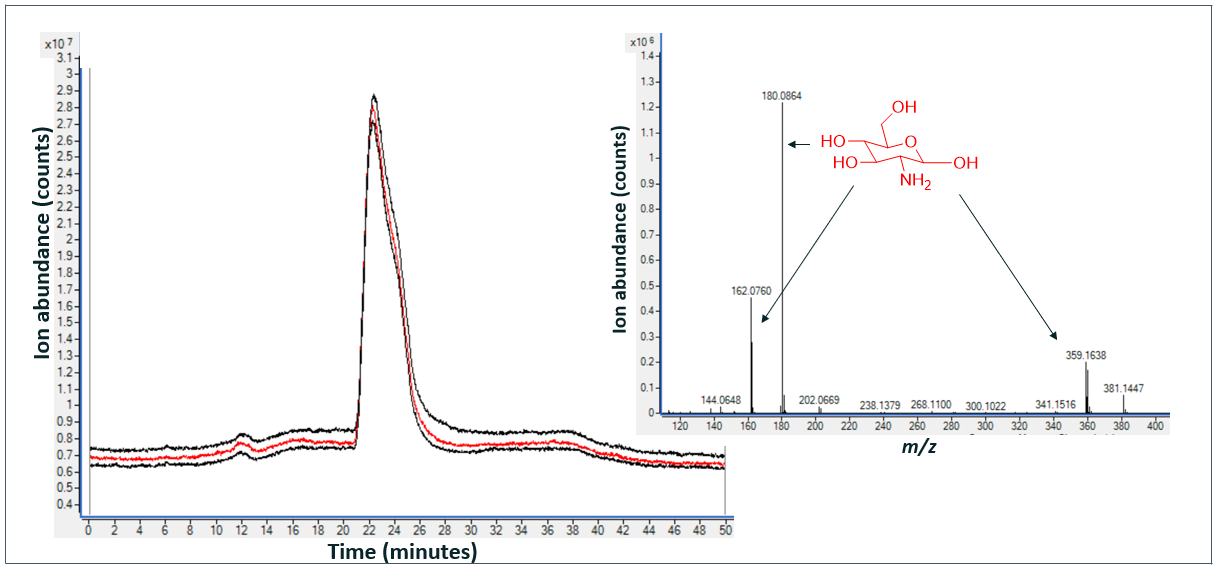


**Fig. S1:** Chromatogram and representative mass spectrum of GlcN standard.

**Mass spectral assignments: Chitin polymer 1 degradation products**


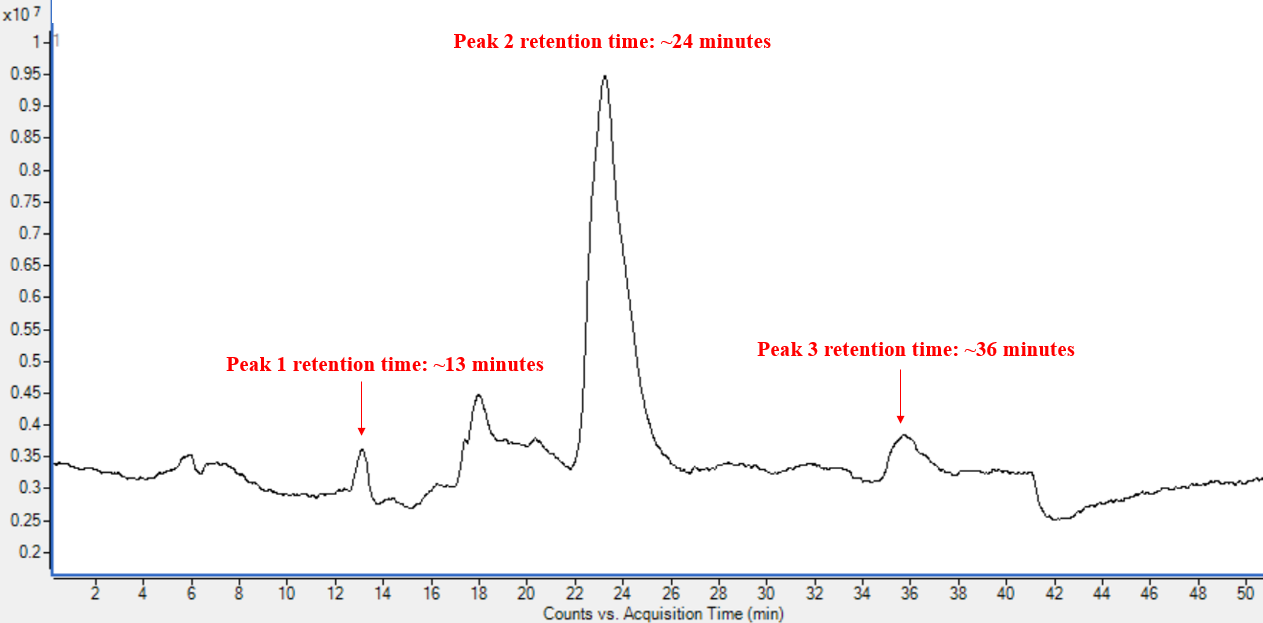


**Fig. S2:** Representative chromatogram of chitin polymer 1 degradation products. Three peaks contained *m/z* signals that indicated the presence of the degradation products of chitin. Unlabeled peaks did not contain signals that correlated to chitin degradation products.

The mass spectrum from the chromatographic peak eluting at 13 minutes contained four signals that were corroborated with GlcNAc adducts, as shown in the mass spectrum in **Figure 5**. The prominent signal at 465 *m/z* is indicative of a compound with a formula matching that of dimerized GlcNAc residues with an associated sodium ion to form a [(2)C_8_H_15_NO_6_ + Na]^+^ adduct. The signal at 244 *m/z* is indicative of an ion with a formula matching that of a sodiated GlcNAc adduct, [C_8_H_15_NO_6_ + Na]^+^. The signal at 222 *m/z* is indicative of a compound with a formula matching that of a protonated GlcNAc adduct, [C_8_H_15_NO_6_ + Na]^+^. The signal at 204 *m/z* is indicative of a compound with a formula matching that of a dehydrated protonated GlcNAc adduct, [C_8_H_15_NO_6_  - H_2_O + H]^+^. Some signals, such as 424 *m/z,* were observed but did not match any predicted or previously observed adducts. These signals may be indictive of unrelated coeluting compounds or of ions that were formed as a result of source fragmentation. Regardless, the presence of multiple adducts provides clear indication of the presence of GlcNAc. Additionally, since this peak is a minor degradation product and would in theory disappear given sufficient polymer exposure to HCl, this was not investigated further.


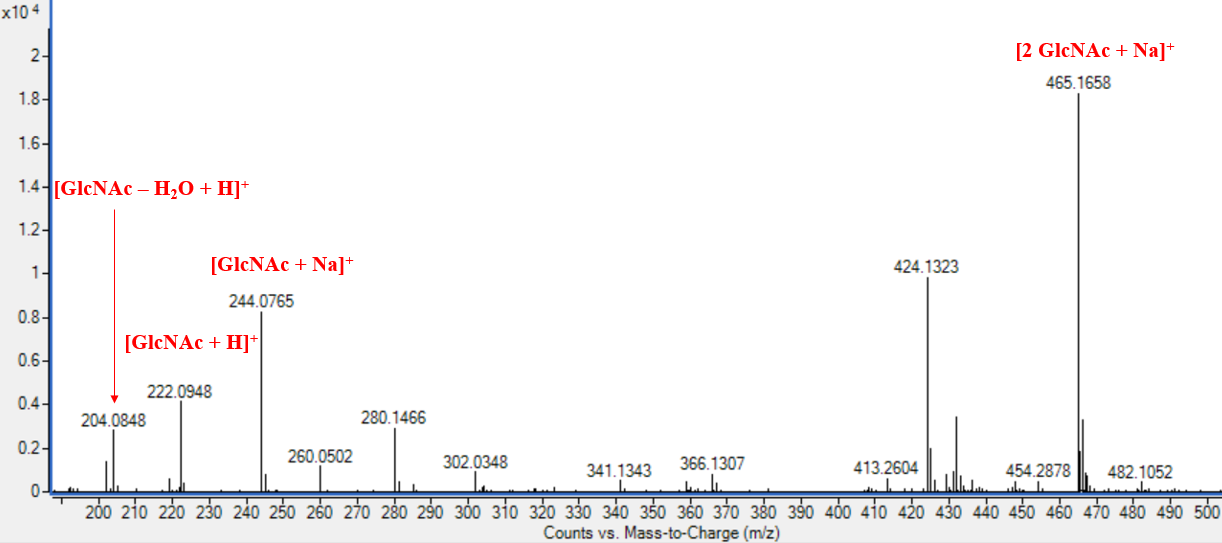


**Fig. S3:** Mass spectrum of the 1^st^ chromatographic peak eluting at 13 minutes. Four signals were identified as *m/z* ratios indicating the presence of compounds with molecular formulas matching those of GlcNAc adducts.

The mass spectrum from the chromatographic peak eluting at 24 minutes contained four signals consistent with GlcN adducts. The prominent signal at 381 *m/z* is indicative of a compound with a formula matching that of GlcN residues that dimerized with a sodium ion to form a [(2) C_6_H_13_NO_5_ + H]^+^ adduct. The signal at 202 *m/z* is indicative of a compound with a formula matching that of a sodiated GlcN adduct, [C_6_H_13_NO_5_ + Na]^+^. The signal at 180 *m/z* is indicative of a compound with a formula matching that of a protonated GlcN adduct, [C_6_H_13_NO_5_ + H]^+^. The signal at 162 *m/z* is indicative of a compound with a formula matching that of a dehydrated and protonated GlcNAc adduct, [C_6_H_13_NO_5_ - H_2_O + H]^+^. Some signals, such as the cluster from 359 - 362 *m/z,* were observed but did not match any predicted or previously observed adducts. A [(2)GlcN + H]^+^ dimerized adduct would result in a signal at 359 *m/z*; however, the isotope pattern of the cluster from 359 – 362 *m/z* indicates that the most abundant species is an isotope at 360 *m/z*. This makes [(2)C_6_H_13_NO_5_ + H]^+^ an unlikely assignment. Interestingly, this cluster was also noted in low abundances in the LC-MS of GlcN standards. The presence of this peak in both the GlcN standard and chitin polymer degradation products provides indication that this signal may represent an unforeseen adduct related to GlcN. There are surprisingly few studies regarding the nature of the ESI ionization of amino sugar residues such as GlcN and GlcNAc. Further investigations into this would provide useful information to define how these molecules behave when ionized.

The mass spectrum from the chromatographic peak eluting at 36 minutes contained three signals consistent with GlcN dimer adducts, as shown in the mass spectrum in **Figure 6**. The prominent signal at 703 *m/z* is indicative of a compound with a formula matching that of two GlcN dimers with an associated sodium ion to form a [(2)C_12_H_24_N_2_O_9_ + Na]^+^ adduct. The signal at 363 *m/z* is indicative of a compound with a formula matching that of a sodiated GlcN dimer, [C_12_H_24_N_2_O_9_ + H]^+^. The signal at 341 *m/z* is indicative of a compound with a formula matching that of a protonated GlcN dimer, [C_12_H_24_N_2_O_9_ + H]^+^.


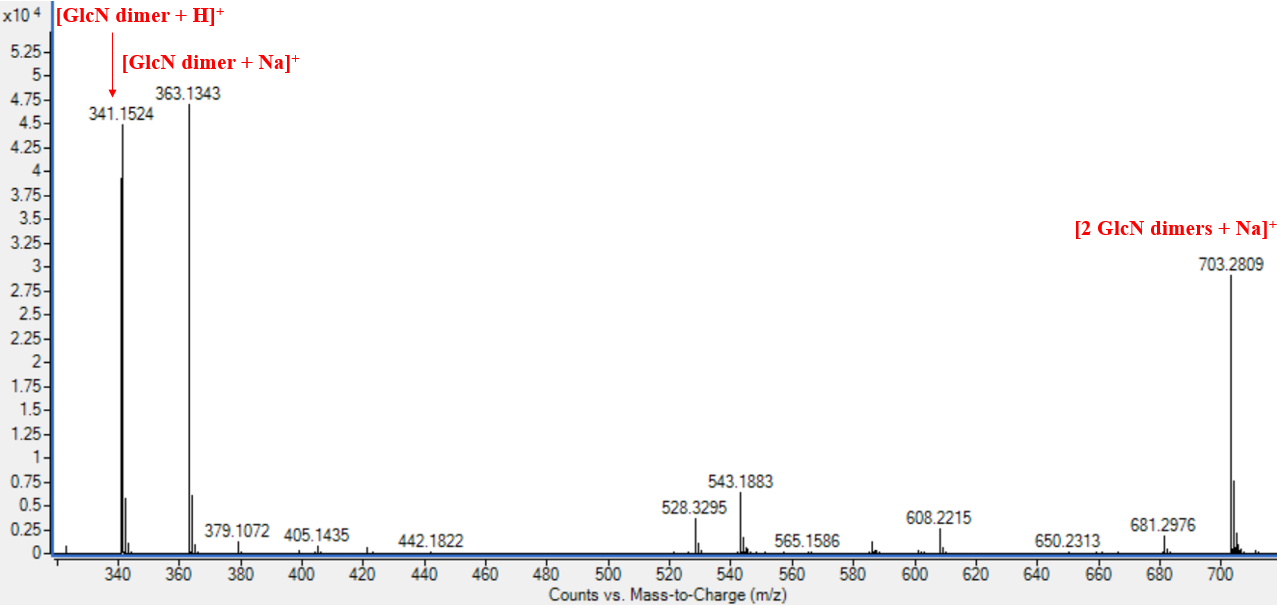
 **Fig. S4:** Mass spectrum of the 3^rd^ chromatographic peak eluting at 36 minutes. Three signals were identified with *m/z* ratios indicating the presence of compounds with molecular formulas matching GlcNAc adducts.

**Mass spectral assignments: Chitin polymer 2 degradation products**


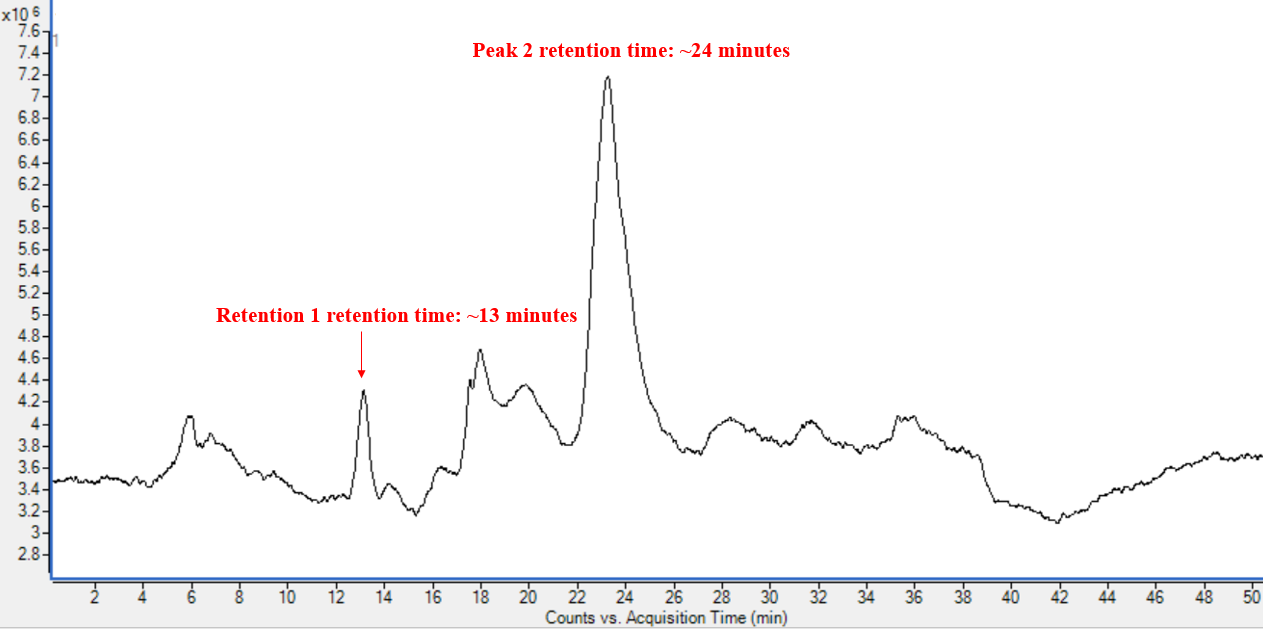


**Fig. S5:** Chromatogram of chitin polymer 2 degradation products. Two peaks contained signals that indicated the presence of chitin degradation products. Remaining peaks did not contain signals representative of chitin degradation products.


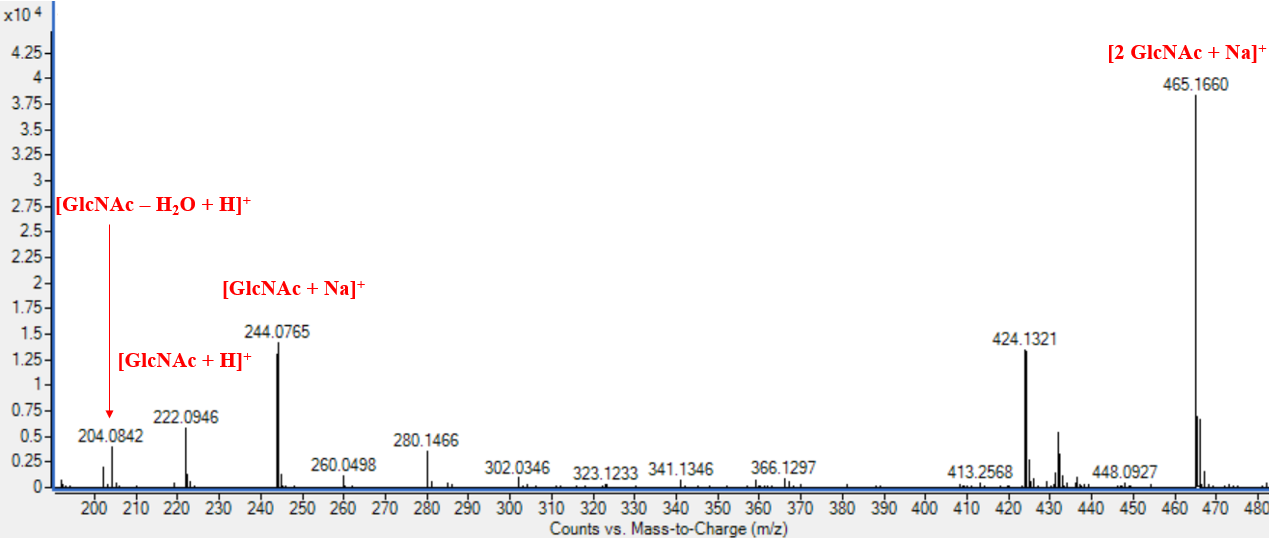


**Fig. S6:** Mass spectrum of the 1^st^ chromatographic peak which eluted at 13 minutes. Four signals indicated the presence of compounds with molecular formulas matching those of GlcNAc adducts.

The mass spectrum from the chromatographic peak eluting at 13 minutes contained four signals consistent with GlcNAc adducts. The prominent signal at 465 *m/z* is indicative of a compound with a formula matching that of dimerized GlcNAc with a sodium ion to form a [(2)C_8_H_15_NO_6_ + Na]^+^ adduct. The signal at 244 *m/z* is indicative of a compound with a formula matching that of a sodiated GlcNAc adduct, [C_8_H_15_NO_6_ + Na]^+^. The signal at 222 *m/z* is indicative of a compound with a formula matching that of a protonated GlcNAc adduct, [C_8_H_15_NO_6_ + Na]^+^. The signal at 204 *m/z* is indicative of a compound with a formula matching that of a dehydrated and protonated GlcNAc adduct, [C_8_H_15_NO_6_  - H_2_O + H]^+^. Once again, the signal at 424 *m/z* discussed earlier was observed.

As observed with the results from chitin polymer 1, the mass spectrum from the chromatographic peak eluting at 24 minutes contained four signals consistent with GlcN adducts. A representative mass spectrum from this peak is shown in **Figure 9**. However, the prominent signal in these spectrum was the species at 180 *m/z,* indicative of a compound with a formula matching that of a protonated GlcN adduct, [C_6_H_13_NO_5_ + H]^+^. The signal at 202 *m/z* is indicative of a compound with a formula matching that of a sodiated GlcN adduct, [C_6_H_13_NO_5_ + Na]^+^. The signal at 162 *m/z* is indicative of a compound with a formula matching that of a dehydrated and protonated GlcNAc adduct, [C_6_H_13_NO_5_ - H_2_O + H]^+^. The signal at 381 *m/z* was observed once again, indicative of a compound with a formula matching that of dimerized GlcN residues with an associated sodium ion to form a [(2) C_6_H_13_NO_5_ + H]^+^ adduct. Additionally, the cluster from 359 - 362 *m/z,* was observed again in mass spectra extracted from this peak.


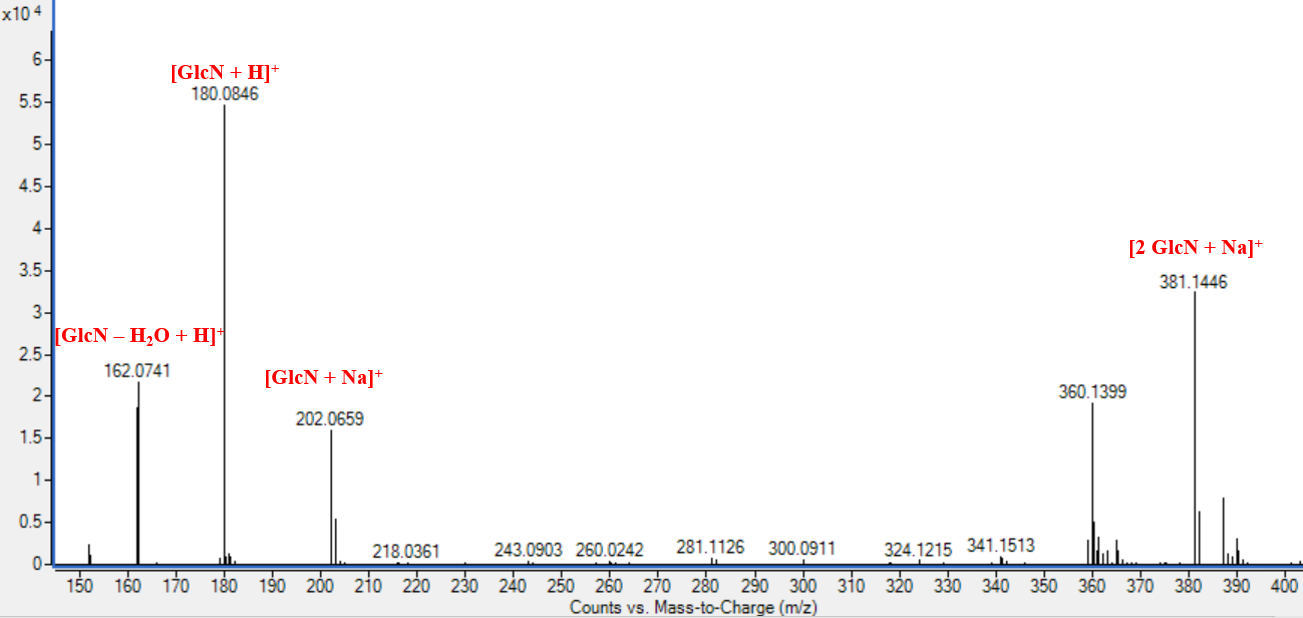


**Fig. S7:** Mass spectrum of the 2^nd^ chromatographic peak eluting at 24 minutes. Four signals were identified with *m/z* ratios indicating the presence of ions with molecular formulas matching GlcN adducts.
